# Diversity and Multiplicity of *P. falciparum* infections among asymptomatic school children in Mbita, Western Kenya

**DOI:** 10.1038/s41598-020-62819-w

**Published:** 2020-04-03

**Authors:** Abdoulie O. Touray, Victor A. Mobegi, Fred Wamunyokoli, Jeremy K. Herren

**Affiliations:** 1 Department of Molecular Biology and Biotechnology, Institute of Basic Sciences, Technology and Innovation, Pan African University (PAUSTI), Nairobi, Kenya; 2 Department of Biochemistry, School of Medicine, University of Nairobi, Nairobi, Kenya; 3 Department of Biochemistry, Jomo Kenyatta University of Agriculture and Technology (JKUAT), Nairobi, Kenya; 4 International Centre of Insect Physiology and Ecology (icipe), Nairobi, Kenya

**Keywords:** Genetics, Molecular biology, Diseases

## Abstract

Multiplicity of infection (MOI) and genetic diversity of *P. falciparum* infections are important surrogate indicators for assessing malaria transmission intensity in different regions of endemicity. Determination of MOI and diversity of *P. falciparum* among asymptomatic carriers will enhance our understanding of parasite biology and transmission to mosquito vectors. This study examined the MOI and genetic diversity of *P. falciparum* parasite populations circulating in Mbita, a region characterized as one of the malaria hotspots in Kenya. The genetic diversity and multiplicity of *P. falciparum* infections in 95 asymptomatic school children (age 5–15 yrs.) residing in Mbita, western Kenya were assessed using 10 polymorphic microsatellite markers. An average of 79.69% (Range: 54.84–95.74%) of the isolates analysed in this study were polyclonal infections as detected in at least one locus. A high mean MOI of 3.39 (Range: 2.24–4.72) and expected heterozygosity (*He*) of 0.81 (Range: 0.57–0.95) was reported in the study population. The analysed samples were extensively polyclonal infections leading to circulation of highly genetically diverse parasite populations in the study area. These findings correlated with the expectations of high malaria transmission intensity despite scaling up malaria interventions in the area thereby indicating the need for a robust malaria interventions particularly against asymptomatic carriers in order to attain elimination in the region.

## Introduction

Malaria continues to be a major public health problem in many parts of the world despite numerous national and international efforts in combatting the ongoing disease transmission. Although enormous success in the fight against the disease has been registered, reports have indicated an insignificant progress in the reduction of global malaria cases for the period 2014–2018^1^. An increase in the global incidence of malaria cases from 219 million in 2017 to 228 million in 2018 with a staggering 405 000 related deaths was registered^1,2^. Sub-Saharan Africa bears the greatest malaria cases and death rates compared to other regions like South-East Asia, Eastern Mediterranean, Western Pacific, and the Americas with an unprecedented 213 million malaria cases^1^.

In Kenya, about 74 percent of the total population are reported to be at risk of malaria infection and regions such as the coastal areas and western part of the country records the highest transmission intensity^3,4^. Children registered the highest prevalence of the disease and recorded an average of 8 percent and 15 percent among younger (<5 years) and older (5–15 years) age categories respectively^5^. Older children living in endemic environments around lake shores recorded a much higher prevalence of about 38 percent^6^. In a bid to attain its goal of a “Malaria-free Kenya”, the country has implemented many malaria control and eradication interventions including; the use of Artemisinin based Combination Therapy (ACT) as a first line of treatment, Long-Lasting Insecticidal Net (LLIN), Intermittent Presumptive Treatment in pregnancy (IPTp), Indoor Residual Spraying (IRS) and Rapid Diagnostic Test (RDT) for efficient parasite diagnosis^7,8^.

A larger proportion of malaria morbidity and mortality studies were centred on preschool-aged children (2–4 years of age). This group contributes a substantial number of incidence to the global malaria burden each year^9–11^. The management and epidemiology of malaria in school-age children as a group has initially received little attention in comparison to the pregnant women and children under the age of five years groups^11–14^. In many endemic settings, malaria contributes immensely to the annual school absenteeism and poor academic performance among school children^9,13,14^. Older children in high malaria transmission areas develop parasite specific immunity against *Plasmodium* parasite and this contributes to reduction in clinical malaria episodes and high prevalence of asymptomatic parasite carriers^15–17^. As a result, malaria asymptomatic school children might not be absent from school due to clinical malaria episodes but may serve as major reservoir for the transmission of the disease^11,18,19^.

Overall, progress towards malaria control and elimination has stalled across all World Health Organisation (WHO) regions. The stagnancy in the global fight against the disease is partly due to decreased funding from international donors^1,20–23^. However, the high prevalence of asymptomatic carriers especially among older children^19,24–28^, high recombination rates among distinct *P. falciparum* clones in endemic settings leading to emergence of highly diverse parasite isolates, rapid emergence and distribution of drug resistant *P. falciparum* parasite strains^29–33^, and prevalence of infections characterized by multiple genetically distinct parasite strains are some of the major contributing factors hindering the global malaria control and elimination^31,34,35^. In highly endemic environments, many individuals carry multiple parasite clones^36–38^. This may have both positive and negative implications in the fight against malaria. The carriage of multiple distinct parasite clones by an individual is reported to enhance the development of multiple strain specific anti-parasite immunity. However, due to an intense intra-host competition, harbouring multiple distinct parasite clones is also implicated in high gametocyte production and emergence of highly virulent and drug resistant parasite strains^32,39–43^.

Multiplicity of infection (MOI) and genetic diversity of *P. falciparum* infection are important surrogate tools for the determination of malaria transmission intensity in different regions of endemicity^44–46^. These indices of *P. falciparum* parasite infections are also used in determining the impact of some key malaria interventions like drug and vaccine efficacy studies^29,47,48^. Numerous studies about multiplicity and diversity of *P. falciparum* infections have been carried out in various WHO regions using different tools including size-polymorphic antigens and microsatellite markers followed by agarose gel and capillary electrophoresis respectively, single nucleotide polymorphism genotyping, amplicon ultra-deep sequencing and whole genome sequencing^36,49^. The use of molecular genotyping of polymorphic antigenic markers like merozoite surface protein (MSP) 1 and 2, glutamate rich protein (Glurp) for parasite diversity studies are faced with some criticisms due to the fact that these genes are implicated to be under strong immune selection^44,50–52^. Amplicon ultra-deep sequencing and whole genome sequencing techniques have high sensitivity and specificity particularly to detect minority clones in case of multiclonal infections^52^. However, the costs and time factors attached to the use of these techniques are relatively high thereby limiting their application especially in resource scarce settings. Microsatellite markers are highly polymorphic, widely distributed in the *Plasmodium* genome, perceived to be selection neutral and readily amplifiable using cheap methods like PCR^35,53,54^. These criteria have made them an ideal tool for the estimation of parasite genetic diversity and MOI^36,46,48^.

While studies about asymptomatic *Plasmodium* parasite carriage among school children have been reported^18,26,28,55^, studies on diversity level and multiplicity of *P. falciparum* infections among this group still remained scanty. This study was carried out to investigate the diversity and multiplicity of *P. falciparum* infections among asymptomatic school children (age 5–15 yrs.) in one of the highly malaria endemic regions of Kenya in order to provide an up-to-date critical information for monitoring parasite transmission dynamics and effective evaluation of the impact of currently implemented malaria control interventions in the area.

## Materials and Methods

### Ethics statement

The Kenya Medical Research Institute (KEMRI) Scientific and Ethics Review Unit (SERU) granted approval for the original study (KEMRI/RES/7/3/1). Consent for sample reuse was part of the original consent form. Study participants were only enrolled after obtaining a written informed consent from their parents or legal guardians. Participants above 12 years also provided assent in addition to parental consent. All experiments were performed in accordance with the relevant guidelines and regulations.

### Study site and sample collection

#### Study site

The study was conducted in Mbita sub-county, situated in the shores of Lake Victoria in Homa Bay County formerly Nyanza Province in western Kenya. The district is bordered from the north, west and south by Lake Victoria and located between latitudes 0° 21′ and 0° 32′ South and longitudes 34° 04′ and 34° 24′ East. The area of the district is about 163.28 km^2^ and is approximately 400 km west of the capital city of Kenya, Nairobi. Mbita sub-county has a population of about 115,896 and the major economic activities include subsistence farming, fishing, small scale businesses and domestication of animals like sheep, goat, chicken, pig and cattle^56^. Majority of the residents live in a compound system which comprises of one or more households together. The housing structures range from traditional mud with grass thatched huts to modern concrete and corrugated iron buildings^56^. The region experiences two periods of rainfall, from March to June and then October to November each year^57^ and is characterized as one of the HIV hotspots with some of the poorest health indicators in Kenya. Due to the biannual rainfall pattern and the proximity to the shores of Lake Victoria, the region experiences a perennial transmission of malaria thereby making the disease the leading cause of morbidity and mortality among children in the region^58^.

#### Sample collection

The study used archived samples that were previously collected from asymptomatic school children in Mbita, western Kenya. The participants were recruited from various primary schools in the region and enrolled between December 2016 and October 2018 as part of a study to evaluate symbiotic microbes and mosquito vector competence using membrane feeding assays in a bid to isolate and characterize potential malaria transmission blocking endosymbiont candidates. Blood samples were obtained from school children aged between 5–15 years and were screened for *Plasmodium* parasite carriage using SD Bioline malaria Ag Pf/Pan (HRP-II/pLDH) Rapid Diagnostic Test (RDT) and microscopy. A drop of blood from each participant was also obtained for the preparation of thick and thin blood films, stained with 10% Giemsa for 10 minutes and used for the microscopy diagnosis. Two drops of blood were collected from each individual on a filter paper (Whatman 3MM; Whatman, Maidstone, United Kingdom), for genomic DNA extraction. The filter paper dry blood spots (DBS) samples were air dried and sealed in airtight plastic bags containing silica gel and were stored at −20 °C until usage. The blood smear slides were read by a well-trained microscopists and any slide with no observable parasites in 100 microscopic fields were declared negative; gametocytes were counted against 500 white blood cells while the asexual parasites were counted against 200 white blood cells. Slides that were declared negative were all double-read and confirmed to be negative if no parasites are observed in 200 microscopic fields. A second microscopist re-read all the slides for the purpose of quality control. A total of 95 RDT and microscopy positive filter paper dry blood spots (DBS) samples were randomly selected for this study.

### Genomic DNA extraction

Single-hole Kangaro punch (No. 376224, India) was used to cut out six circles (3 mm in diameter) from each filter paper dry blood spots (DBS) sample and the punched DBS were placed in 1.5 ml Eppendorf tubes. In order to ensure that there is no cross contamination between samples, the punch was sterilized using sodium hypochlorite (NaClO) and 100% ethanol^59^.

DNA was extracted using the QIAamp DNA Mini Kit (51304, QIAGEN, Hilden, Germany) following the manufacturer’s protocol. The quality and concentration of each extracted genomic DNA sample was assessed using a Nanodrop 2000C (Thermo Fisher Scientific, Waltham, MA, USA) and samples were stored at −20 °C.

### Microsatellite genotyping of *P. falciparum*

*Plasmodium falciparum* DNA extracted from each DBS sample was genotyped using ten polymorphic microsatellite markers^60^. Primer sets targeting specific sequences flanking (TAA)n repeat regions in the *P. falciparum* genome were used to amplify each locus using a slightly modified published hemi-nested PCR protocol in a SimpliAmp Thermal Cycler (Applied Biosystems, Loughborough, UK)^60^. The primers were dye labelled using 6-FAM for locus TA1, ARA2, PfPK2, TA87, and TA109; HEX for TA81, TA42, Polyα, TA60 and Pfg377. The hemi-one amplifications were performed in a total PCR reaction volume of 20 µL containing 1× FIREPol Master Mix (Solis BioDyne, Estonia), 0.3 µM of each primer (Macrogen, South Korea) and 3 µL (10 ng/µL) of DNA template. The cycling conditions for nest-one PCR were; initial denaturation for 2 min at 94 °C; 30 cycles of 30 sec at 94 °C, 30 sec at 42 °C, 30 sec at 40 °C and 30 sec at 65 °C; final elongation for 5 min at 65 °C. The total reaction volume of hemi-two PCR was also 20 µL containing 1× FIREPol Master Mix (Solis BioDyne, Estonia), 0.4 µM of each primer (Macrogen, South Korea) (see Supplementary Table S1) and 5 µL of hemi-one PCR amplicons. The PCR conditions were as follows; initial denaturation for 2 min at 94 °C; 30 cycles of 30 sec at 94 °C, 30 sec at 45 °C and 30 sec at 65 °C; final elongation for 5 min at 65 °C.

The PCR amplification for the different loci were confirmed by fragment separation in 2% agarose gels and visualized using ethidium bromide (E1385, Merck, Germany). The amplified products were pooled together as described elsewhere^54^, wrapped in an aluminium foil then shipped for fragment analysis at the DNA Sequencing Facility, University of Illinois at Urbana-Champaign, Urbana, Illinois, USA. The pooled PCR products were separated on an ABI 3730XL (Applied Biosystems) genetic Analyser using GeneScan 400HD ROX Size Standard (Applied Biosystems, Foster City, CA). Allele sizes scoring and peak height quantification was carried out using GeneMarker V3.0.1 software (SoftGenetics, LLC).

### Data analysis

The fact that *Plasmodium falciparum* parasite stages found in human blood are haploid, the presence of more than one allele at a particular locus per isolate was interpreted as polyclonal or multiple clone infections. That is, samples with more than one allele at any given locus are termed as co-infection with genetically distinct parasite clones. Isolates with minor allele peak heights that are ≥25% of the predominant allele per locus were scored as multiple alleles. DNA samples with only one allele detected at all the genotyped gene loci were scored as monoclonal infections. Multiplicity of *P. falciparum* infection (MOI) is defined as the number of genetically distinct clones co-infecting a host at any given time. In this study, MOI and average MOI were estimated per locus. The average MOI of all the samples included in this study was estimated as the ratio of the total number of distinct fragments (genetically distinct parasite clones) scored for a given gene loci in relation to the number of samples successfully amplified by that marker locus^35^.

Descriptive statistics were computed using IBM SPSS Statistics for Windows, version 20 (IBM Corp., Armonk, N.Y., USA). Allelic frequency, expected heterozygosity, number of alleles and number of effective alleles of the study population were determined using a population genetic software, GenAIEx 6.5^61^. The predominant allele in case of multiple alleles or the only allele in the case of single allele at each locus was used to estimate the expected heterozygosity (*H*_*e*_) per locus as a measure of genetic diversity using a formula, *H*_*e*_ = [n/(n − 1)][1 −Σp_i_^2^], where n and p_i_ represents the number of isolates analysed and frequency of the i^th^ allele in a given population. Expected heterozygosity (*H*_*e*_) values range between 0 and 1 for no genetic diversity and high genetic diversity, respectively^60^.

## Results

### Characteristics of study participants

A total of 95 *P. falciparum* positive samples detected by RDT and microscopy was included in this study. The ages of the study participants ranged from 5 to 15 years. In Table 1, the total number of participants within the age categories 5–9 and 10–15 years were 59 (62.1%) and 36 (37.9%), respectively. The female participants comprised of 42 (44.2%) while 53 (55.8%) were of the male gender. Based on the RDT and microscopy results (data not shown), 77 (81.1%) of the participants were infected with only *P. falciparum* while 18 (18.9%) of the subjects were infected with *P. falciparum* plus another *Plasmodium* species (*P. ovale and P. malariae*) and 50.5% carried the sexual stage of the parasite (gametocyte).

**Table 1:** Demographic characteristics of study subjects.

Variables	Number of samples (N)	Percent (%)
**Age (years)**		
5–9	59	62.1
10–15	36	37.9
Total	95	100
**Sex**		
Female	42	44.2
Male	53	55.8
Total	95	100
**Malaria Infection**		
Single	77	81.1
Mixed	18	18.9
Total	95	100
**Microscopy**		
Asexual	47	49.5
Gametocyte	48	50.5
Total	95	100

### Multiple clone infections and multiplicity of *P. falciparum* infection (MOI)

**Figure 1:**
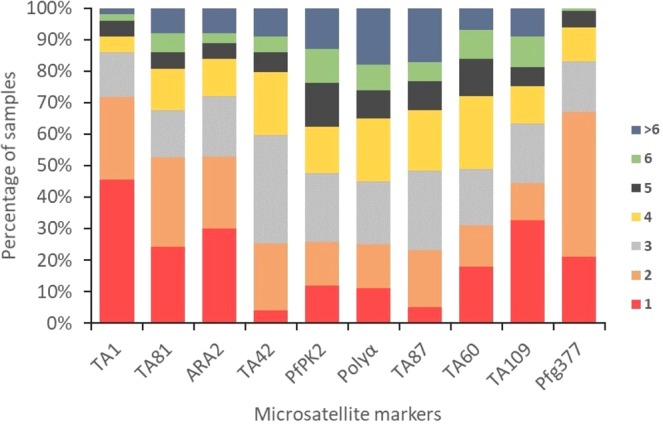
Frequency of samples with different number of alleles detected in the study at each of the 10 genotyped *P. falciparum* microsatellite loci. The various colours in the graph representing the different number of clones: red (1), orange (2), gray (3), gold (4), black (5), green (6), and dark blue (>6).

The amplification of the 10 polymorphic microsatellite marker loci was successful in 99% of the isolates. Overall, the Pfg377 locus was the marker with the highest number of missing data in which 3 (3%) of the isolates had a missing data. The full genotype profile and allelic frequencies of the 95 samples were presented as Supplementary Tables S2 and S3, respectively. For this study, samples were grouped into two categories depending on the number of alleles detected per marker loci. Isolates with only one allele per marker locus were classified as monoclonal while those with two or more alleles at either one or more loci were termed polyclonal infections. The percentage of polyclonal infections in the study samples was computed based on the proportion of isolates with multiple genotypes per marker. In Fig. 1 and Table 2, samples analysed in this study contained largely polyclonal parasites [79.69% (Range: 54.84–95.74%)] with high multiplicity of *P. falciparum* infections [3.39 (Range: 2.24–4.72)] detected at each of the genotyped genetic locus. More than six alleles were found at almost all the genotyped genetic loci (except in the Pfg377 loci) in the study samples indicating an overall complex infections.

**Table 2:** Characteristics of the 10 microsatellite markers.

Marker loci	No. Positive (%)^a^	Allelic range (bp)	Number of Monoclonal infections (%)^b^	Number of Polyclonal infections (%)^c^	Mean MOI
TA1	93 (98)	159–204	42 (45.16)	51 (54.84)	2.24
TA81	95 (100)	112–142	23 (24.21)	72 (75.79)	3.05
ARA2	93 (98)	63–90	28 (30.11)	65 (69.89)	2.84
TA42	94 (99)	182–248	4 (4.26)	90 (95.74)	3.55
PfPK2	93 (98)	159–192	11 (11.83)	82 (88.17)	4.04
Polyα	95 (100)	114–198	10 (10.53)	85 (89.47)	4.72
TA87	95 (100)	90–123	5 (5.26)	90 (94.74)	4.22
TA60	94 (99)	69–99	17 (18.09)	77 (81.91)	3.63
TA109	94 (99)	154–208	31 (32.98)	63 (67.02)	3.27
Pfg377	92 (97)	89–113	19 (20.65)	73 (79.35)	2.37
Mean			**20.31%**	**79.69%**	**3.393**

^a^Number of samples successfully amplified by each locus. ^b^Number of monoclonal infections is the number of isolates with only one allele score per locus. ^c^Number of polyclonal infections is the number of isolates with more than one allele score per locus.

Overall, based on genotyping of the 10 polymorphic microsatellite marker loci, there were variations in the number of isolates with polyclonal infections detected by each marker locus with TA1 (54.84%) and TA42 (95.74%) markers detecting the lowest and highest number of polyclonal isolates (Fig. 1 and Table 2).

### Genetic diversity of *P. falciparum* infections in the study population

The 10 microsatellite marker loci used in this study were all observed to be highly polymorphic in the study population with total number of distinct observed alleles (Na) per locus ranging from 8 (for locus Pfg377) to 23 (for locus Polyα) and the number of effective alleles (Ne) varied between 2.3 and 15.3. The least and most polymorphic markers were Pfg377 and Polyα respectively. Overall, Na values were much higher than the Ne values indicating that majority of the alleles were present at low frequency (Table 3 and Supplementary Fig. S1 and Table S3).

**Table 3:** Genetic diversity of the 95 *Plasmodium falciparum* samples based on 10 microsatellite markers.

Locus	N	Allelic Range (bp)	Na	Ne	*He*	Mean MOI
TA1	93	159–204	15	6.469	0.855	2.240
TA81	95	112–142	10	4.593	0.791	3.050
ARA2	93	63–90	9	6.327	0.851	2.840
TA42	94	182–248	18	10.298	0.913	3.550
PfPK2	93	159–192	11	9.076	0.899	4.040
Polyα	95	114–198	23	15.323	0.945	4.720
TA87	95	90–123	11	8.318	0.889	4.220
TA60	94	69–99	11	6.094	0.845	3.630
TA109	94	154–208	10	2.278	0.567	3.270
Pfg377	92	89–113	8	2.360	0.583	2.370
Average			12.600	7.114	0.814	3.393
SD			4.695	3.905	0.133	0.780
SE			1.485	1.235	0.042	0.253

*N* = Number of samples amplified; *Na* = Number of Different Alleles; *Ne* = Number of Effective Alleles = 1/(∑p_i_^2^); *He* = Unbiased Diversity/expected heterozygosity = [n/(n − 1)][1 −Ʃp_i_^2^]; *MOI* = multiplicity of infection. *Na*, *Ne* and *He* were calculated from the predominant allele data set while *Mean MOI* scores per locus were calculated by dividing the sum of all the detected alleles at each individual locus by the number of samples positive for that given marker locus.

The expected heterozygosity (*He*) defined as the probability that two randomly selected clones from a population will carry distinct alleles at each marker loci was used to estimate the allelic diversity per loci based on allelic frequencies computed (See Supplementary files: Table S3) at each of the 10 microsatellite loci. In Table 3, the expected heterozygosity *(He)* values per locus in the study population varied from 0.57 (TA109) to 0.95 (Polyα) with an average score of 0.81, thereby depicting a high genetic diversity among the isolates.

## Discussion

The current study revealed a high level of multiplicity of infections and genetic diversity among the study population. This is illustrated by high level of polyclonal infections and high proportions of locus scores with multiple genotypes. The 79.69% prevalence of polyclonal infections found in the study population is consistent with results from other studies carried out in malaria endemic and high transmission intensity regions where multi-clonal infection rates varied from 50% to 100%, respectively^62–67^. The overall mean MOI of 3.39 in the study population found in our study corroborates with other studies in high malaria transmission areas in Africa like Kenya (2.83)^68^, Gambia (2.1), Senegal (2.2), Guinea Bissau (2.6), Guinea (4.0)^54^, Tanzania (3.5)^62^, Uganda (3.0–3.7)^65^ and Madagascar (3.72–3.73)^66^ but higher than results from low malaria transmission regions like Djibouti (1.0–1.4), Dakar (1.0–1.5), Niamey (1.0–1.8)^67^ and Sudan^69^. The high MOI found in this study maybe due to the boosted parasite specific immunity in people residing in high malaria transmission regions and the study population as children were reported to carry higher MOI infections compared to adults^38,70^.

The high number of alleles and high expected heterozygosity of *P. falciparum* recorded at each of the 10 polymorphic microsatellite marker loci in our study are indications of high genetic diversity among *P. falciparum* parasites circulating in the study area. This finding could be due to the high malaria transmission and endemicity reported in the area^71^. Another contributing factor for the high genetic diversity maybe due to the efficient land and water transport networks in the study area facilitating human movements from other malaria endemic regions leading to the introduction of foreign strains to the local parasites gene pool^64,68^. These results corroborates the findings of previous studies conducted in other parts of western Kenya^63,65,66^ but different from those of low malaria transmission intensity areas like lowland coastal area of Kenya (Malindi)^64^, Philippines^72^ and other regions of South America^73–75^. The high mean *He* value (0.81) observed in our study was similar to those obtained in high malaria endemic and transmission settings of Africa: Kenya (0.81)^64^, Congo DRC (0.71)^75^, Ghana (0.67–0.69)^35^, Nigeria (0.65–0.79)^76^, The Gambia (0.75), Senegal (0.72), Guinea Bissau (0.78), Guinea Conakry (0.78)^54^, Asia: Vietnam (0.52–0.91) and Oceania: Papua New Guinea (0.79)^73^. However, *He* values from studies conducted in low malaria transmission intensity regions such as Djibouti (0.41)^67^, Brazil (0.39–0.52)^77^, Honduras (0.35) and Nicaragua (0.38)^78^ were lower than that of our study.

## Conclusions

The analysis of genetic diversity and multiplicity of *P. falciparum* infections among malaria asymptomatic school children (age 5–15 yrs.) in Mbita, western Kenya using 10 polymorphic microsatellite marker loci reveal a high level of multi-clonal infections and high genetic diversity of *P. falciparum* parasites circulating in the study area. These findings correlated with the expectations of high malaria transmission intensity and endemic region despite the scaling up of malaria control interventions by the government. Therefore, in order to attain ultimate malaria elimination in the region, there is a need for the implementation of more robust and holistic interventions against the parasite particularly the asymptomatic carriers. Furthermore, the findings from our study have pinpointed the need for periodic *Plasmodium* parasite molecular genetic surveillance studies in the area. This will help maintain up-to-date information about parasite transmission dynamics for effective impact assessment of the currently implemented malaria control interventions. Additionally, it will further guide in the introduction of new malaria control programs whenever needed.

## Supplementary information

Supplementary Information.

Supplementary Information 2.
