# Supplementary material for: Diversity and Multiplicity of P. falciparum infections among asymptomatic school children in Mbita, Western Kenya
Source: Sci Rep. 2020 Apr 3;10:5924. doi: 10.1038/s41598-020-62819-w (PMC7125209; doi:10.1038/s41598-020-62819-w)
Supplement: Supplementary file 1 — Supplementary Information. [file 41598_2020_62819_MOESM1_ESM.pdf]

Diversity and Multiplicity of *P. falciparum* infections among asymptomatic school children in Mbita, Western Kenya

Abdoulie O. Touray[1*], Victor A. Mobegi[2*], Fred Wamunyokoli[3], Jeremy K. Herren[4]

[1] Department of Molecular Biology and Biotechnology, Institute of Basic Sciences, Technology and Innovation, Pan African University (PAUSTI), Nairobi, Kenya; abdoulietouray79@gmail.com (A.O.T.)

[2] Department of Biochemistry, School of Medicine, University of Nairobi, Nairobi, Kenya; vatunga@uonbi.ac.ke (V.A.M.)

[3] Department of Biochemistry, Jomo Kenyatta University of Agriculture and Technology (JKUAT), Nairobi, Kenya; fwamunyokoli@jkuat.ac.ke (F.W.)

[4] International Centre of Insect Physiology and Ecology (*icipe*), Nairobi, Kenya; jherren@icipe.org (J.K.H.)

***** Correspondence: vatunga@uonbi.ac.ke; abdoulietouray79@gmail.com

Supplementary Figure

The figure below is available as supplementary figure,

Figure S1: Allelic diversity at the 10 microsatellite loci markers computed from the allelic frequencies data.

Figure S1. Allelic diversity at the 10 microsatellite loci markers computed from the allelic frequencies data. Allelic frequencies were generated from the predominant allele data using GeneMarker V3.0.1 (SoftGenetics, LLC).

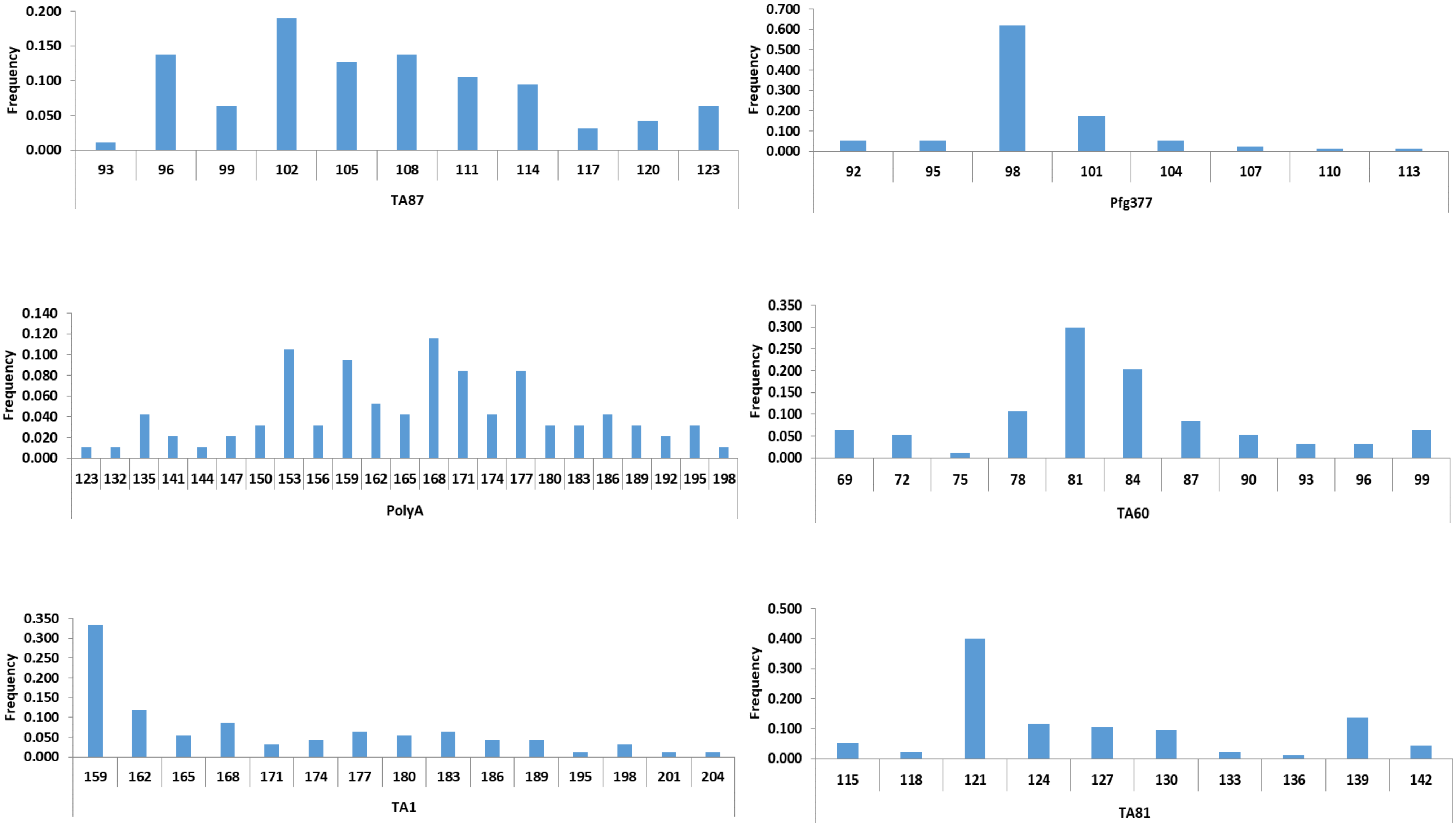

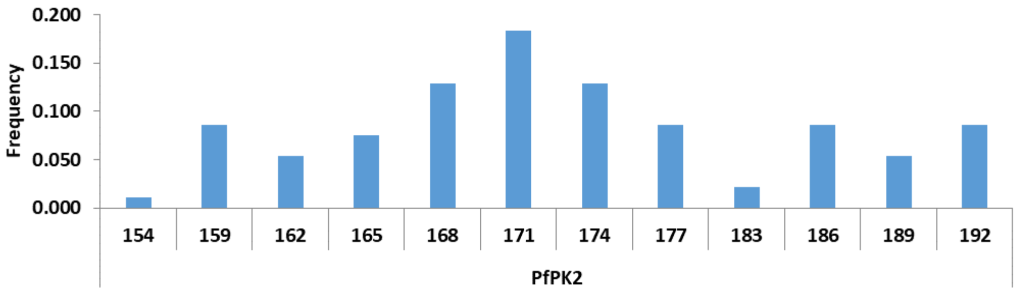
Frequency
0.200
0.150
0.100
0.050
0.000
154 159 162 165 168 171 174 177 183 186 189 192
PfPK2

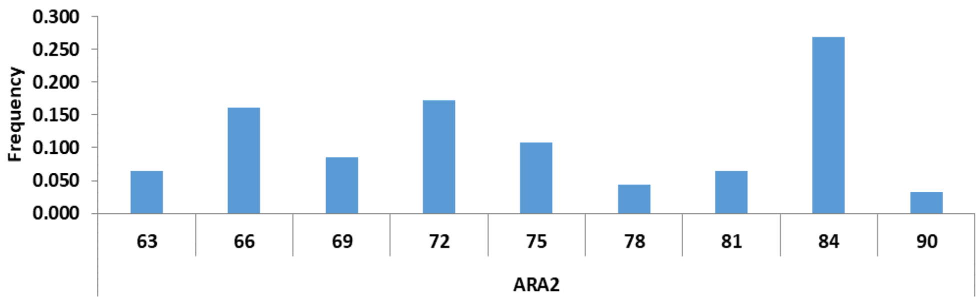
Frequency
0.300
0.250
0.200
0.150
0.100
0.050
0.000
63 66 69 72 75 78 81 84 90
ARA2

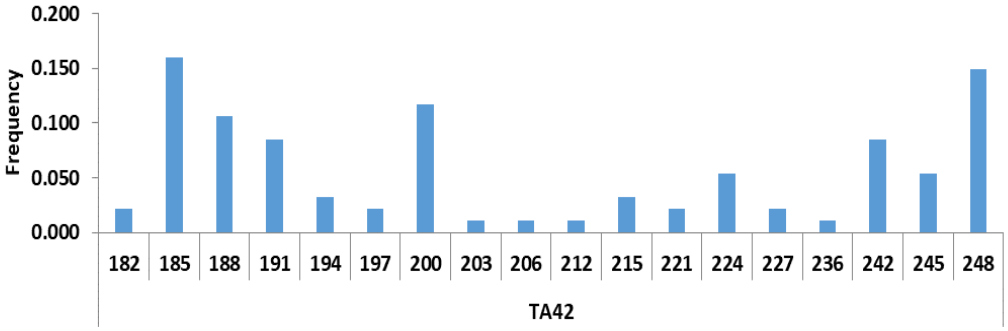
Frequency
0.200
0.150
0.100
0.050
0.000
182 185 188 191 194 197 200 203 206 212 215 221 224 227 236 242 245 248
TA42

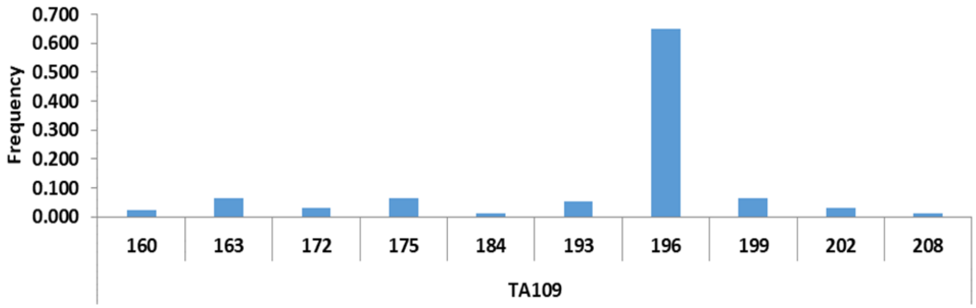
Frequency
0.700
0.600
0.500
0.400
0.300
0.200
0.100
0.000
160 163 172 175 184 193 196 199 202 208
TA109
