# Supplementary material for: Diversity and Multiplicity of P. falciparum infections among asymptomatic school children in Mbita, Western Kenya
Source: Sci Rep. 2020 Apr 3;10:5924. doi: 10.1038/s41598-020-62819-w (PMC7125209; doi:10.1038/s41598-020-62819-w)
Supplement: Supplementary file 2 — Supplementary Information 2. [file 41598_2020_62819_MOESM2_ESM.pdf]

Diversity and Multiplicity of *P. falciparum* infections among asymptomatic school children in Mbita, Western Kenya

Abdoulie O. Touray[1*], Victor A. Mobegi[2*], Fred Wamunyokoli[3], Jeremy K. Herren[4]

[1] Department of Molecular Biology and Biotechnology, Institute of Basic Sciences, Technology and Innovation, Pan African University (PAUSTI), Nairobi, Kenya; abdoulietouray79@gmail.com (A.O.T.)

[2] Department of Biochemistry, School of Medicine, University of Nairobi, Nairobi, Kenya; vatunga@uonbi.ac.ke (V.A.M.)

[3] Department of Biochemistry, Jomo Kenyatta University of Agriculture and Technology (JKUAT), Nairobi, Kenya; fwamunyokoli@jkuat.ac.ke (F.W.)

[4] International Centre of Insect Physiology and Ecology (*icipe*), Nairobi, Kenya; jherren@icipe.org (J.K.H.)

* Correspondence: vatunga@uonbi.ac.ke; abdoulietouray79@gmail.com

Supplementary Tables

The following four tables below are available as supplementary tables,

Table S1: Microsatellite Genotyping Primer list.

Table S2: Alleles scored in each of the 95 *P. falciparum* isolates genotyped at 10 microsatellite loci.

Table S3: Allele frequencies at 10 microsatellite loci in the 95 *Plasmodium falciparum* samples.

Table S1: Microsatellite Genotyping Primer list

Loci	Primer Name	Primer sequence (5' to 3')	Chromosome Number	Dye label	Accession number	size range from Anderson 1999
**TA1**	*TA1-3(F)_a*	CTACATGCCTAATGAGCA	6	FAM	AF010507	159-204
	TA1-R_b	TTTTATCTTCATCCCCAC				
	TA1-F_c	CCGTCATAAGTGCAGAGC				
**TA81**	*TA81-3(F)_a*	GAAGAAATAAGGGAAGGT	5	HEX	AF010510	112-142
	TA81-R_b	TTTCACACAACACAGGATT				
	TA81-F_c	TGGACAAATGGGAAAGGATA				
**ARA2**	*ARA2-3(F)_a*	GTACATATGAATCACCAA	11	FAM	X17484	63-90
	ARA2-R_b	GCTTTGAGTATTATTAATA				
	ARA2-F_c	GAATAAACAAAGTATTGCT				
**TA42**	*TA42-3(F)_a*	ACAAAAGGGTGGTGATTCT	5	HEX	AF010543	182-251
	TAA42-R_b	GTATTATTACTACTACTAAAG				
	TAA42-F_c	TAGAAACAGGAATGATACG				
**PfPK2**	*PfPK2-3(R)_a*	CCTCAGACTGAAATGCAT	12	FAM	X63648	159-192
	PfPK2-F_b	CTTTCATCGATACTACGA				
	PfPK2-R_c	AAAGAAGGAACAAGCAGA				
**Polyα**	*Polyα-R_a*	ATCAGATAATTGTTGGTA	4	HEX	L18785	114-201
	Polyα-F_b	AAAATATAGACGAACAGA				
	Polyα-3(IR)_c	GAAATTATAACTCTACCA				
**TA87**	*TA87-3(F)_a*	ATGGGTTAAATGAGGTACA	6	FAM	AF010571	90-126
	TAA87-R_b	ACATGTTCATATTACTCAC				
	TAA87-F_c	AATGGCAACACCATTCAAC				
**TA60**	*TA60-F_a*	CTCAAAGAAAAATAATTCA	13	HEX	AF010556	69-99
	TAA60-R_b	AAAAAGGAGGATAAATACAT				
	TAA60-3(IF)_c	TAGTAACGATGTTGACAA				
**TA109**	*TA109-3(F)_a*	TAGGGAACATCATAAGGAT	6	FAM	AF010508	154-223
	TAA109-R_b	CCTATACCAAACATGCTAAA				
	TAA109-F_c	GGTTAAATCAGGACAACAT				
**Pfg377**	*Pfg377-3(R)_a*	TTATGTTGGTACCGTGTA	12	HEX	L04161	89-113
	Pfg377-F_b	GATCTCAACGGAAATTAT				
	Pfg377-R_c	TTATCCCTACGATTAACA				

Table S2: Alleles scored in each of the 95 *P. falciparum* isolates genotyped at 10 microsatellite loci.

		Marker Loci									
No.	**Sample ID**	**TA1**	**TA81**	**ARA2**	**TA42**	**PfPK2**	**PolyA**	**TA87**	**TA60**	**TA109**	**Pfg377**
**1**	AL002-NA-25.fsa	159	139	72	191	168	174	111	78	196	98
**2**	AL003-NA-34.fsa	159	130	72	188	159	123	96	87	196	98
**3**	AL017-NA-44.fsa	159	121	66	191	186	186	108	0	196	101
**4**	AL050-NA-30.fsa	0	121	78	248	168	186	108	81	196	107
**5**	AL054-NA-76.fsa	186	130	72	194	174	159	114	81	202	101
**6**	AL055-NA-89.fsa	189	121	75	185	162	150	108	90	196	98
**7**	AL061-NA-60.fsa	186	118	75	185	177	159	105	84	196	98
**8**	AL064-NA-6.fsa	159	127	84	248	186	168	108	81	163	98
**9**	AL065-NA-12.fsa	177	121	63	224	168	171	120	72	193	101
**10**	AL090-NA-50.fsa	174	130	69	191	174	153	108	84	175	101
**11**	AL106-NA-23.fsa	180	124	84	200	174	177	102	90	196	98
**12**	AL121-NA-9.fsa	168	121	72	185	159	180	99	78	163	98
**13**	GA012-NA-78.fsa	189	139	84	248	171	183	96	87	196	98
**14**	GA013-NA-75.fsa	159	121	81	188	171	156	96	99	196	98
**15**	GA020-NA-67.fsa	174	139	66	212	177	183	111	84	196	104

**16**	GA034-NA-5.fsa	165	130	84	242	186	168	108	81	196	98
**17**	GA039-NA-36.fsa	165	115	69	185	165	165	123	81	0	113
**18**	GA044-NA-93.fsa	198	121	72	188	159	153	99	84	160	104
**19**	GA046-NA-48.fsa	159	124	84	245	192	168	105	81	196	98
**20**	GE022-NA-45.fsa	180	121	72	248	159	153	105	84	160	98
**21**	GE023-NA-80.fsa	159	121	75	242	171	174	111	90	196	92
**22**	GE024-NA-56.fsa	162	121	72	200	186	162	114	69	196	101
**23**	GE035-NA-61.fsa	171	121	81	200	171	177	105	84	163	101
**24**	KE002-NA-71.fsa	159	124	66	242	177	171	114	69	175	98
**25**	KE003-NA-41.fsa	159	139	63	245	159	162	96	81	196	98
**26**	KE018-NA-49.fsa	198	115	75	248	192	165	105	69	196	98
**27**	KE023-NA-62.fsa	177	121	66	248	0	153	120	78	196	95
**28**	KI003-NA-29.fsa	171	121	78	185	159	159	96	81	196	95
**29**	KI004B-NA-86.fsa	162	121	90	215	162	135	105	87	196	0
**30**	KI004-NA-64.fsa	162	121	84	194	177	162	117	87	202	92
**31**	KI006-NA-26.fsa	159	121	75	194	174	195	96	96	196	98
**32**	KI016B-NA-47.fsa	159	121	84	200	174	198	102	81	196	0

**33**	KI016-NA-27.fsa	159	124	84	185	168	159	105	81	196	104
**34**	KI045-NA-35.fsa	159	139	84	197	171	135	99	84	196	98
**35**	KI047-NA-66.fsa	162	133	84	203	168	186	105	84	199	101
**36**	KI053-NA-65.fsa	177	130	84	191	168	183	111	96	202	92
**37**	KI064-NA-40.fsa	159	139	90	248	168	189	105	84	196	101
**38**	KM002A-NA-31.fsa	177	121	66	242	189	150	93	93	172	95
**39**	KM002B-NA-21.fsa	159	121	66	188	171	180	96	99	196	92
**40**	KM019-NA-69.fsa	159	142	84	191	162	168	111	81	196	101
**41**	KM028-NA-83.fsa	198	115	69	185	192	153	99	99	196	98
**42**	KM046-NA-18.fsa	183	121	84	188	168	141	123	78	163	98
**43**	KM046B-NA-88.fsa	183	115	84	185	165	144	96	81	163	98
**44**	KM061-NA-68.fsa	0	133	75	206	192	171	111	84	196	104
**45**	KMSB017-NA-84.fsa	168	139	72	224	192	150	123	78	193	98
**46**	KMSB020-NA-4.fsa	168	124	84	248	189	168	114	84	208	98
**47**	KS016-NA-95.fsa	162	121	66	215	186	168	102	84	175	98
**48**	KS020-NA-59.fsa	180	142	81	188	171	165	114	84	199	98
**49**	KS022-NA-53.fsa	162	136	84	191	168	147	96	99	196	98

**50**	KS045-NA-52.fsa	186	121	84	221	177	177	114	84	199	92
**51**	KS047-NA-77.fsa	159	124	90	188	165	135	102	90	196	98
**52**	KT005-NA-87.fsa	180	127	72	248	171	156	102	84	175	98
**53**	KT020-NA-22.fsa	168	121	72	185	171	174	108	75	196	101
**54**	KT033-NA-38.fsa	162	121	78	191	159	162	108	87	196	98
**55**	MI008-NA-73.fsa	159	121	69	182	0	192	105	93	196	98
**56**	MI009-NA-57.fsa	174	121	72	242	177	177	99	81	196	98
**57**	MI022-NA-91.fsa	168	115	84	236	186	159	96	81	184	101
**58**	MI024-NA-63.fsa	183	121	81	215	171	171	120	69	196	98
**59**	MI033-NA-43.fsa	159	121	72	185	192	186	102	87	199	107
**60**	MI034-NA-13.fsa	168	118	72	248	183	171	108	90	196	98
**61**	ALR026-NA-28.fsa	171	124	84	242	165	168	117	81	199	101
**62**	NY001-NA-20.fsa	159	139	66	227	192	153	108	93	196	0
**63**	NY002-NA-3.fsa	159	121	66	245	159	162	105	81	196	98
**64**	NY004-NA-11.fsa	189	121	66	185	168	135	96	99	196	104
**65**	NY005-NA-2.fsa	165	121	66	182	189	156	108	81	196	98
**66**	NY013-NA-10.fsa	162	139	63	200	165	168	96	84	172	98

**67**	NY021-NA-79.fsa	195	121	75	0	177	177	120	72	196	98
**68**	NY025-NA-32.fsa	183	127	84	248	171	195	102	72	196	98
**69**	NY026-NA-24.fsa	204	139	75	197	171	171	102	84	196	95
**70**	NY039-NA-15.fsa	159	139	84	185	168	153	108	81	196	95
**71**	NY043-NA-81.fsa	177	127	72	200	189	180	117	81	196	98
**72**	NY047-NA-7.fsa	159	121	69	224	174	159	108	87	196	101
**73**	NY050-NA-54.fsa	159	124	63	200	162	171	102	69	196	98
**74**	NY066-NA-46.fsa	174	121	69	248	183	165	111	99	196	101
**75**	NY080-NA-16.fsa	159	121	0	200	165	195	102	78	196	98
**76**	NY087-NA-33.fsa	159	130	75	242	174	168	108	81	196	98
**77**	NY093-NA-17.fsa	165	142	63	224	174	141	102	81	193	98
**78**	OS016-NA-19.fsa	159	139	66	224	192	153	123	84	196	98
**79**	OS050-NA-37.fsa	183	127	81	242	171	189	102	78	193	98
**80**	OS060-NA-94.fsa	162	127	84	188	171	189	102	78	193	98
**81**	OS062-NA-1.fsa	168	142	66	185	159	153	111	81	163	98
**82**	OS068-NA-39.fsa	165	121	81	188	186	159	114	84	196	101
**83**	OS069-NA-72.fsa	159	127	84	227	171	132	114	72	175	98

**84**	OS073-NA-8.fsa	186	127	75	185	165	177	102	69	196	98
**85**	OS098-NA-14.fsa	159	121	63	191	174	153	111	87	196	98
**86**	OS115-NA-42.fsa	183	127	84	248	189	192	102	72	196	98
**87**	OS123-NA-82.fsa	162	124	84	200	174	174	99	81	175	98
**88**	OS149-NA-74.fsa	189	124	69	245	171	171	105	81	196	110
**89**	OS152-NA-58.fsa	201	130	72	221	174	147	102	81	199	101
**90**	OS169-NA-85.fsa	162	121	66	200	171	168	123	81	196	98
**91**	OS180-NA-92.fsa	180	130	69	185	168	168	102	81	196	98
**92**	OS184-NA-55.fsa	177	127	72	245	186	177	105	78	196	98
**93**	PW087-NA-90.fsa	159	139	0	248	162	177	96	96	196	98
**94**	SA022-NA-70.fsa	168	124	66	200	177	159	99	78	196	98
**95**	SA027-NA-51.fsa	159	130	78	188	174	159	114	81	172	98

Highlighted yellow are the predominant alleles within mixed genotype infections while single unmixed alleles are not highlighted. Missing genotype data is reflected by zero.

Table S3: Allele frequencies at 10 microsatellite loci in the 95 *Plasmodium falciparum* samples

**Locus**	**Allele**	
**TA1**	n	93
	159	0.333
	162	0.118
	165	0.054
	168	0.086
	171	0.032
	174	0.043
	177	0.065
	180	0.054
	183	0.065
	186	0.043
	189	0.043
	195	0.011
	198	0.032
	201	0.011
	204	0.011
**TA81**	n	95
	115	0.053
	118	0.021
	121	0.400
	124	0.116
	127	0.105
	130	0.095
	133	0.021
	136	0.011
	139	0.137

	142	0.042
**ARA2**	n	93
	63	0.065
	66	0.161
	69	0.086
	72	0.172
	75	0.108
	78	0.043
	81	0.065
	84	0.269
	90	0.032
**TA42**	n	94
	182	0.021
	185	0.160
	188	0.106
	191	0.085
	194	0.032
	197	0.021
	200	0.117
	203	0.011
	206	0.011
	212	0.011
	215	0.032
	221	0.021
	224	0.053
	227	0.021
	236	0.011
	242	0.085

	245	0.053
	248	0.149
**PfPK2**	n	93
	159	0.097
	162	0.054
	165	0.075
	168	0.129
	171	0.183
	174	0.129
	177	0.086
	183	0.022
	186	0.086
	189	0.054
	192	0.086
**Polyα**	n	95
	123	0.011
	132	0.011
	135	0.042
	141	0.021
	144	0.011
	147	0.021
	150	0.032
	153	0.105
	156	0.032
	159	0.095
	162	0.053
	165	0.042
	168	0.116

	171	0.084
	174	0.042
	177	0.084
	180	0.032
	183	0.032
	186	0.042
	189	0.032
	192	0.021
	195	0.032
	198	0.011
**TA87**	n	95
	93	0.011
	96	0.137
	99	0.074
	102	0.179
	105	0.137
	108	0.147
	111	0.095
	114	0.095
	117	0.032
	120	0.042
	123	0.053
**TA60**	n	94
	69	0.064
	72	0.053
	75	0.011
	78	0.106
	81	0.298

	84	0.202
	87	0.085
	90	0.053
	93	0.032
	96	0.032
	99	0.064
**TA109**	n	94
	160	0.021
	163	0.064
	172	0.032
	175	0.064
	184	0.011
	193	0.053
	196	0.649
	199	0.064
	202	0.032
	208	0.011
**Pfg377**	n	92
	92	0.054
	95	0.054
	98	0.620
	101	0.174
	104	0.054
	107	0.022
	110	0.011
	113	0.011
